# Understanding the medication regimens associated with anticholinergic burden in older people's mental health services in the UK

**DOI:** 10.1192/bjo.2024.788

**Published:** 2024-10-03

**Authors:** Thomas R. E. Barnes, Delia Bishara, Alistair Burns, Phyo K. Myint, Olivia Rendora, Elena M. Edokpolor Pernia, Carol Paton

**Affiliations:** Division of Psychiatry, Imperial College London, London, UK; Prescribing Observatory for Mental Health, Royal College of Psychiatrists, London, UK; Institute of Psychiatry, Psychology and Neuroscience, King's College London, London, UK; Mental Health of Older Adults and Dementia, South London and Maudsley NHS Foundation Trust, London, UK; Division of Neuroscience, University of Manchester, Manchester, UK; Ageing Clinical & Experimental Research (ACER) Team, Institute of Applied Health Sciences, University of Aberdeen, Aberdeen, UK

**Keywords:** Anticholinergic, dementia, older adults, mental health

## Abstract

**Background:**

Medications with anticholinergic properties are associated with a range of adverse effects that tend to be worse in older people.

**Aims:**

To investigate medication regimens with high anticholinergic burden, prescribed for older adults under the care of mental health services.

**Method:**

Clinical audit of prescribing practice, using a standardised data collection tool.

**Results:**

Fifty-seven trusts/healthcare organisations submitted data on medicines prescribed for 7915 patients: two-thirds (66%) were prescribed medication with anticholinergic properties, while just under a quarter (23%) had a medication regimen with high anticholinergic burden (total score ≥3 on the anticholinergic effect on cognition (AEC) scale). Some 16% of patients with a diagnosis of dementia or mild cognitive impairment were prescribed medication regimens with a high anticholinergic burden, compared with 35% of those without such diagnoses. A high anticholinergic burden was mostly because of combinations of commonly prescribed psychotropic medications, principally antidepressant and antipsychotic medications with individual AEC scores of 1 or 2.

**Conclusions:**

Adults under the care of older people's mental health services are commonly prescribed multiple medications for psychiatric and physical disorders; these medication regimens can have a high anticholinergic burden, often an inadvertent consequence of the co-prescription of medications with modest anticholinergic activity. Prescribers for older adults should assess the anticholinergic burden of medication regimens, assiduously check for adverse anticholinergic effects and consider alternative medications with less anticholinergic effect where indicated. The use of a scale, such as the AEC, which identifies the level of central anticholinergic activity of relevant medications, can be a helpful clinical guide.

Medications with antimuscarinic properties (hereafter referred to as ‘anticholinergics’) block the effects of the neurotransmitter, acetylcholine, in the brain and peripheral nervous system. Central anticholinergic effects include cognitive impairment, delirium, behavioural disturbances, anxiety and insomnia, as well as poorer outcomes for psychotic illness.^1–3^ Peripheral adverse effects include dry mouth, constipation, blurred vision, urinary retention and falls.^4^ The long-term use of such medications has been found to be associated with worsening cognitive function and an increased incidence of dementia,^2,5–9^ as well as poorer physical function, acute cardiovascular events, problems such as pneumonia and decreased lung function and increased all-cause mortality.^10–13^

## Anticholinergic burden

The cumulative anticholinergic effect of all the medications prescribed for a person is referred to as the anticholinergic burden. Older adults are more likely to be exposed to a high anticholinergic burden, as they are more commonly prescribed multiple medications with anticholinergic properties to treat comorbid medical conditions.^14–16^ For those patients under the care of older people's mental health services, several such medicines are likely to be started, including antidepressants and antipsychotic medications, as well as antimuscarinic agents. These patients may also be prescribed medicines with anticholinergic properties by other medical services, for conditions such as unstable bladder, gastro-intestinal symptoms, neuropathic pain, Parkinson's disease and chronic obstructive airways disease.

## Prescribing medications with anticholinergic effects

Guidelines have recommended limiting the anticholinergic burden for older adults,^17^ which means prescribing medication regimens with less anticholinergic effect.^18,19^ However, such a strategy depends on a knowledge of the medications responsible for the cumulative burden for any individual patient, and in any particular clinical service, so that suitable alternatives can be selected. In the context of a quality improvement programme addressing the use of anticholinergic (antimuscarinic) medications, developed by the Prescribing Observatory for Mental Health (POMH), such information was collected in a clinical audit of prescribing practice in older people's mental health services.

## Method

In 2023, POMH conducted a clinical audit of prescribing practice in older people's mental health services in the UK, focusing on the use of medications with anticholinergic properties. All 66 POMH member trusts/healthcare organisations were invited to take part. All the clinical services participating in the audit used a standardised, bespoke data collection tool designed to capture information from the clinical records that related to clinical performance against several practice standards. These were partly derived from National Institute for Health and Care Excellence (NICE) guidelines NG123^20^ and NG97;^21^ both of these guidelines had full patient and public involvement. Further, the standards were endorsed by an expert advisory group (D.B., A.B., P.K.M.). This paper reports on data relating to two of the practice standards: (1) when prescribing an antidepressant or antipsychotic medication in older people, a medication with a low/no anticholinergic burden should be considered and (2) when a medication is prescribed for urinary incontinence in older people, a medication with a low/no anticholinergic burden should be considered.

In each participating trust and healthcare service, data on relevant clinical practice were collected by clinicians, pharmacists and clinical audit staff from the clinical records of a sample of patients under the care of older people's mental health services. These data were pseudonymous within the trusts but submitted anonymously to POMH, using Formic software (version 5.7.1 for Windows, Formic Healthcare; https://www.formic.com/ (note: this URL is for a website with information about the product but a subscription is required to obtain it)).^22^ The key fields in the data collection tool were mandatory, preventing incomplete submissions. Ethical approval was not required for such an audit-based quality improvement initiative.^23^

The data were analysed using SPSS (version 26.0 for Windows, IBM; https://www.ibm.com/support/pages/downloading-ibm-spss-statistics-26),^24^ principally to determine the extent to which the performance of each participating trust complied with the practice standards, although these findings are not presented here.

The anticholinergic effect on cognition (AEC) scale^25^ allocates a score of 0–3 for each of the medications included, based on the strength of their antimuscarinic effects and penetration across the blood–brain barrier. The AEC scale was used to identify the central anticholinergic effect of all the medications with anticholinergic properties that were prescribed for the patients in the sample.

## Results

Fifty-seven National Health Service (NHS) trusts/healthcare organisations submitted data on the medicines prescribed for 7915 patients under the care of older people's mental health services. The demographic and clinical characteristics of these patients are shown in Table 1. There was a range of psychiatric diagnoses but most of the patients (*n* = 4836, 61%) had either a dementia diagnosis (*n* = 4081) or mild cognitive impairment (MCI) (*n* = 135), or were currently being assessed for dementia (*n* = 620), and were considered together as a ‘dementia subgroup’. The majority (80.5%, *n* = 6372) of the patients were resident in their own home or in a residential or nursing home, and almost all the others were in-patients (19%, *n* = 1511) on a mental health or acute hospital ward.

**Table 1:** Demographic characteristics and clinical characteristics of the 7915 patients under the care of older people's mental health services in the total audit sample

Key demographic variables and clinical setting			Total national sample *n* = 7915
Gender: *n* (%)	Female		4606 (58)
	Male		3309 (42)
Ethnicity: *n* (%)	White/White British		6389 (81)
	Asian/Asian British		218 (3)
	Black/Black British		171 (2)
	Mixed or other		165 (2)
	Other – Chinese		15 (<1)
	Not collected/stated/refused		957 (12)
Age in years: median (IQR)			79 (11)
Clinical psychiatric diagnoses (ICD-10 codes): *n* (%)	Mental and behavioural disorders owing to psychoactive substance use (F10–F19)		241 (3)
	Schizophrenia, schizotypal, delusional disorders and other non-mood psychotic disorders (F20–F29)		846 (11)
	Mood (affective) disorders (F30–F39)	Bipolar disorder (F31)	529 (7)
		Other affective disorder (F30, F32–F39)	1958 (25)
	Anxiety, dissociative, stress-related, somatoform and other nonpsychotic mental disorders (F40–F48)		997 (13)
	Behavioural syndrome associated with physiological disturbance and physical factors (F50–F59)		160 (2)
	Disorders of adult personality and behaviour (F60–F69)		166 (2)
	Intellectual disability/mental retardation (F70–F79)		43 (1)
	Other psychiatric diagnosis		187 (2)
	No psychiatric diagnosis/unclear		3765 (48)
Dementia diagnoses (ICD codes): *n* (%)	Dementia in Alzheimer's disease (F00, G30)		1980 (25)
	Mixed dementia (F00.2, G30.8)		858 (11)
	Vascular dementia (F01)		551 (7)
	Unspecified dementia (F03)		254 (3)
	Other dementia diagnoses		1193 (15)

IQR, interquartile range; ICD-10, International Statistical Classification of Diseases and Related Health Problems, 10th Revision.

### Antidepressant medication

Just over half (*n* = 4240, 54%) of the patients in the total audit sample were prescribed antidepressant medication and, for 16% of these patients, this had a cumulative AEC score of 2 or more. The most commonly prescribed antidepressant medication with an AEC score of 3 was amitriptyline. Table 2 lists all of the antidepressant medications that were prescribed for at least 1% of the patients on such medication, and their AEC scores.

**Table 2:** Antidepressant and antipsychotic medications prescribed for at least 1% of patients in the relevant subsamples

Medications prescribed	AEC score	National subsample *n* (%)	Dementia subgroup *n* (%)	
			Yes	No
**Antidepressants**		*n* = 4240	*n* = 2273	*n* = 1967
Mirtazapine	1	1744 (41)	832 (37)	912 (46)
Sertraline	1	1230 (29)	714 (31)	516 (26)
Venlafaxine	0	591 (14)	164 (7)	427 (22)
Citalopram	1	363 (9)	233 (10)	130 (7)
Amitriptyline	3	262 (6)	150 (7)	112 (6)
Trazodone	0	249 (6)	213 (9)	36 (2)
Duloxetine	0	235 (6)	86 (4)	149 (8)
Fluoxetine	1	205 (5)	96 (4)	109 (6)
Vortioxetine	0	87 (2)	30 (1)	57 (3)
Escitalopram	1	57 (1)	20 (1)	37 (2)
Paroxetine	2	51 (1)	31 (1)	20 (1)
Clomipramine	3	23 (1)	6 (<1)	17 (1)
**Antipsychotic medication**		*n* = 3142	*n* = 1461	*n* = 1681
Regular oral antipsychotic medication				
Risperidone	0	827 (26)	629 (43)	198 (12)
Olanzapine	2	709 (23)	215 (15)	494 (29)
Quetiapine	2	542 (17)	260 (18)	282 (17)
Aripiprazole	1	490 (16)	196 (13)	294 (17)
Amisulpride	0	82 (3)	40 (3)	42 (2)
Haloperidol	0	79 (3)	45 (3)	34 (2)
Clozapine	3	68 (2)	27 (2)	41 (2)
Flupentixol	1	41 (1)	5 (<1)	36 (2)
Paliperidone	1	17 (1)	3 (<1)	14 (1)
Zuclopenthixol	1	17 (1)	2 (<1)	15 (1)
Regular long-acting injectable antipsychotic medication				
Flupentixol decanoate	1	116 (4)	22 (2)	94 (6)
Aripiprazole	1	71 (2)	10 (1)	61 (4)
Paliperidone	1	69 (2)	14 (1)	55 (3)
Zuclopenthixol decanoate	1	67 (2)	15 (1)	52 (3)
Risperidone	0	26 (1)	10 (1)	16 (1)
Haloperidol decanoate	0	16 (1)	4 (<1)	12 (1)

AEC, anticholinergic effect on cognition.

More than one antidepressant medication was prescribed for 890 (21%) of these patients, with 110 (12%) of these combinations having a total AEC score of 4 or more. The antidepressant medications most often prescribed as part of such combinations were mirtazapine (*n* = 740, 83%), venlafaxine (*n* = 334, 38%) and sertraline (*n* = 272, 31%). Of the patients in the dementia subgroup, 6.5% (*n* = 314) were prescribed more than one antidepressant, while the respective proportion for those not in the subgroup was 19% (*n* = 576) (χ^2^ = 281.2, d.f. = 1, *P* < 0.001).

### Antipsychotic medication

Two-fifths (*n* = 3142, 40%) of the national sample were prescribed antipsychotic medication; the respective proportion for those patients in the dementia subgroup was just under a third (1461 of 4836, 30%) and for those not in the subgroup it was just over half (1681 of 3079, 55%) (χ^2^ = 467.3, d.f. = 1, *P* < 0.001). Oral risperidone was the most commonly prescribed antipsychotic medication for those patients in the dementia subgroup: 13% (629 out of 4836) compared with 6% (198 out of 3097) of those not in the subgroup (χ^2^ = 86.9, d.f. = 1, *P* < 0.001).

Table 2 shows the antipsychotic medications prescribed for at least 1% of the patients on such treatment, and their AEC scores. Some 157 (5%) patients were prescribed more than one antipsychotic medication, most commonly a combination of a long-acting injectable (LAI) and oral formulation, which in 13 (8%) patients resulted in an antipsychotic regimen with a total AEC score of 4 or more.

Of the 1461 patients in the dementia subgroup prescribed antipsychotic medication, the most documented target symptoms/behaviours were agitation (*n* = 656, 45%), known or suspected psychotic symptoms (*n* = 542, 37%), physical aggression (*n* = 429, 29%), verbal aggression (*n* = 395, 27%) and distress (*n* = 315, 22%).

### Anticholinergic (antimuscarinic) agents

Anticholinergic (antimuscarinic) agents were prescribed for 289 (4%) patients in the total audit sample (see Table 3). The medications most prescribed were procyclidine (*n* = 222, 77%) and hyoscine (*n* = 55, 19%): for the former, in 169 (76%) patients, the documented rationale for its use was the treatment of extrapyramidal symptoms (EPSs), while the main reason for using the latter was to treat hypersalivation (*n* = 35, 64%). Of the patients prescribed procyclidine, 44 (20%) were co-prescribed an antipsychotic medication with an AEC score of 2 or more, principally olanzapine or quetiapine.

**Table 3:** Anticholinergic (antimuscarinic) medications and medications for urinary incontinence/bladder instability, prescribed for at least 1% of the patients in the relevant subsamples

Medications prescribed	AEC score	National subsample *n* (%)	Dementia subgroup *n* (%)	
			Yes	No
**Anticholinergic medications**		*n* = 289	*n* = 103	*n* = 186
Procyclidine	3	222 (77)	70 (68)	152 (82)
Hyoscine hydrobromide	3	55 (19)	26 (25)	29 (16)
Trihexyphenidyl (benzhexol)	3	9 (3)	3 (3)	6 (3)
Glycopyrronium bromide	1	4 (1)	3 (3)	1 (1)
Atropine drops sublingually or as mouthwash	1	3 (1)	3 (3)	−
Orphenadrine	3	2 (1)	1 (1)	1 (1)
**Urinary incontinence/bladder instability medications**		*n* = 399	*n* = 232	*n* = 167
Mirabegron	0	165 (41)	100 (43)	65 (39)
Solifenacin	1	137 (34)	80 (34)	57 (34)
Oxybutynin	3	44 (11)	24 (10)	20 (12)
Tolterodine	2	38 (10)	25 (11)	13 (8)
Trospium chloride	0	18 (5)	9 (4)	9 (5)
Fesoterodine fumarate	0	6 (2)	5 (2)	1 (1)
Darifenacin hydrobromide	0	5 (1)	2 (1)	3 (2)

AEC, anticholinergic effect on cognition scale.

### Medications for urinary incontinence/bladder instability

A prescription for a medication for urinary incontinence/bladder instability was identified in the clinical records of 399 patients (5%) in the total national sample (see Table 3). The proportion of patients prescribed such medication in the dementia subgroup (*n* = 232, 5%) was the same as for that not in the dementia subgroup (*n* = 167, 5%).

### Other medications with anticholinergic properties

Other medications with anticholinergic properties, not accounted for above, were prescribed for 1251 (16%) patients in the total patient sample. The three most frequently prescribed medicines in this group were diazepam (*n* = 358, 29%), lithium (*n* = 273, 22%) and promethazine (*n* = 230, 18%). Regarding medications for physical illness (other than treatments for urinary incontinence/bladder instability, see above), the most commonly prescribed was prednisolone (*n* = 102, 1% of the total patient sample), which has an AEC score of 1.

### Total anticholinergic burden

**Fig. 1:**
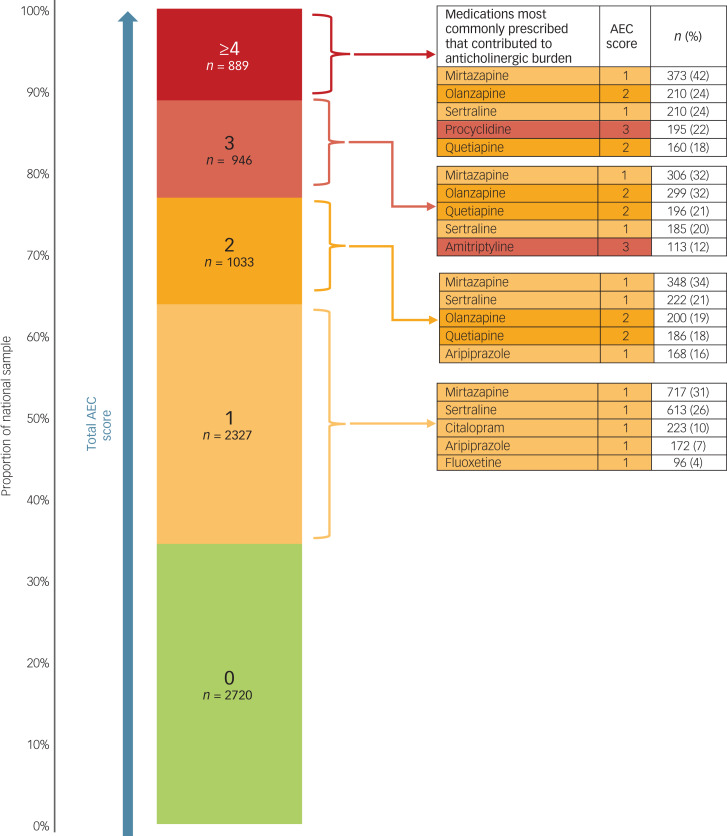
Distribution of the total anticholinergic effect on cognition (AEC) scores of medication regimens prescribed for the patients in the total audit sample and the most prescribed medications contributing to the anticholinergic burden.

Figure 1 shows that, of the total national sample of patients, two-thirds (*n* = 5195; 66%) were prescribed medication with anticholinergic properties. For just under a quarter (*n* = 1835: 23%), the total AEC score for their medication regimen was 3 or more, indicative of a high anticholinergic burden which, in 71% (*n* = 1304) of such patients was because of a combination of medicines with AEC scores of 1 or 2. As can be seen in Fig. 1, a relatively small number of commonly prescribed psychotropic medications was implicated in most patients.

Of the 4836 patients in the dementia subgroup, 16% (*n* = 769) were prescribed a medication regimen with a total AEC score of 3 or more, while for the 3079 patients who were not in the dementia subgroup, the respective proportion was 35% (*n* = 1066) (χ^2^ = 370.2, d.f. = 1, *P* < 0.001).

## Discussion

The main study finding is that medications with anticholinergic properties are commonly prescribed in older people's mental health services. These are predominantly psychotropic medications such as antidepressants and antipsychotics, pharmacological treatments for urinary incontinence and bladder instability and anticholinergic agents used to treat conditions such as EPSs and hypersalivation. Medications for physical health problems, other than those prescribed for urinary incontinence/bladder instability, made a minimal contribution to anticholinergic burden.

For nearly a quarter of the patients in this large audit sample, the medication regimen had a total anticholinergic burden score of 3 or more on the AEC scale. This reflects high central anticholinergic activity that is likely to impact negatively on cognitive and physical function, and therefore be clinically relevant. In line with previous findings, such scores were predominantly because of combinations of medicines with AEC scores of 1 or 2:^16,26^ a relatively small number of commonly prescribed psychotropic medications were implicated in the majority of patients. This suggests that many of the patients were inadvertently exposed to a high anticholinergic burden.

The use of medication with anticholinergic properties is not recommended in older adults, partly because of the association with cognitive decline and incident dementia,^7,10,27,28^ which may be dose dependent,^9^ and partly because such patients may have a poorer prognosis in terms of mortality and risk of admission to hospital if exposed to a high anticholinergic burden.^29^ The findings from the audit suggest that, in clinical practice, there is some avoidance of such medication in those patients with evidence of cognitive decline, in that those patients with a diagnosis of dementia, or MCI or currently being assessed for dementia were much less likely to have a medication regimen with a high level of anticholinergic burden.

A medication regimen with an AEC score of 3 or more is an indication that a medication review is warranted, with consideration being given to withdrawal of the culpable medications and switching to safer alternatives.^29,30^ However, the extent to which such interventions in older people can safely and effectively reduce anticholinergic burden and, consequently, maintain or improve cognitive function, remains to be convincingly demonstrated.^19,31,32^

### Antidepressant medication

Just over half of the patients in the total audit sample were taking antidepressant medication. However, such medication may be less effective in the treatment of depression in older patients when there is comorbid dementia^33^ or other cognitive deficits^34^ and the evidence for efficacy and safety in the treatment of symptoms of behavioural and psychological symptoms associated with dementia (BPSD) is inconsistent and uncertain.^35–38^ Further, these medicines can cause several side effects to which older people are more vulnerable; for example, selective serotonin reuptake inhibitors (SSRIs) are associated with hyponatremia,^39^ reduced bone mineral density,^40^ falls^41^ and gastro-intestinal bleeds.^42^

By far the most prescribed antidepressants were mirtazapine and sertraline; these medications are generally well tolerated by the elderly, with mirtazapine often being selected when insomnia is particularly problematic and sertraline when there is comorbid cardiac pathology.^43^ While both mirtazapine and sertraline have a relatively low AEC score of 1, they commonly contributed to medication regimens with a high total anticholinergic burden. Amitriptyline was the single most commonly prescribed medication, with an AEC score of 3. This medication is often used to treat neuropathic pain, which is likely to have been the indication in many patients.

Those patients in the dementia subgroup were less likely to have been prescribed an antidepressant than those without evidence of dementia, and the choice of antidepressant differed between these two clinical subgroups: trazodone (AEC score of 0) was prescribed slightly more often for the former while mirtazapine (AEC score of 1) and venlafaxine (AEC score of 0) were prescribed more often for the latter.

### Antipsychotic medication

Antipsychotic medication was commonly prescribed in the audit sample, but for fewer patients in the dementia subgroup compared with those not in the subgroup. As might be expected, given its licensed indications and an AEC score of 0, risperidone was the most prescribed antipsychotic medication for those with evidence of dementia but also for the total sample overall. The next most prescribed antipsychotic medications were olanzapine and quetiapine, both of which have an AEC score of 2 and commonly contributed to medication regimens with a high total anticholinergic burden.

A range of antipsychotic medications were prescribed for the patients in the dementia subgroup, principally to manage BPSD, although only haloperidol and risperidone are licensed for this indication. NICE supports such use but only when other non-pharmacological interventions have failed/are not appropriate, symptoms are severe and the patient is at risk of harm and is distressed.^21^ This recommendation is based on the known risk–benefit balance of these medicines for the treatment of BPSD: the effect size is small and overall mortality is increased,^44,45^ mostly because of an increased risk of stroke. NICE has estimated that for every 1000 patients with BPSD who take antipsychotic medication for 12 weeks, 12 would have a stroke that would not have occurred otherwise and 11 would die who would not have done so otherwise.^46^

### Anticholinergic (antimuscarinic) agents

Anticholinergic (antimuscarinic) agents were prescribed for less than 4% of the total audit sample. NICE advises that all such medications should be prescribed with caution in the elderly,^20,21^ particularly those with dementia. Procyclidine and hyoscine hydrobromide, both medications with AEC scores of 3, accounted for the vast majority of prescriptions.

Procyclidine was predominantly used to treat EPSs, principally Parkinsonism, but also for a few instances of tardive dyskinesia, for which it is not considered to be effective and may even worsen symptoms.^47,48^ Given that procyclidine is used specifically for its anticholinergic effects, strategies to reduce its use might start with consideration of the potential for intervening earlier in the prescribing cascade, that is, by optimising the dose of antipsychotic medication and considering whether it would be feasible to switch to another antipsychotic with a lower propensity to cause EPSs.

Hyoscine hydrobromide was mostly used to treat hypersalivation. A variety of antimuscarinic agents, including hyoscine and sublingual atropine,^49,50^ are commonly prescribed for hypersalivation associated with clozapine. However, there is a lack of consensus about efficacy in this context,^51,52^ so they should be prescribed for each patient as an individual treatment trial.

### Medications for urinary incontinence/bladder instability

In the total national sample, 5% of patients were identified as being prescribed medication for the management of overactive bladder/urinary incontinence. The findings suggest that pre-existing cognitive impairment does not influence the choice of medication: three-quarters of those in both the dementia and no dementia clinical subgroups were prescribed either mirabegron or solifenacin, which have AEC scores of 0 and 1, respectively. This may reflect that most prescribers of such medication are aware that NICE recommends minimisation of the anticholinergic burden associated with these medicines in older adults.^20,53^ Nevertheless, of the medications prescribed for urinary problems, oxybutynin (AEC score of 3) was chosen for more than 10% of patients and tolterodine (AEC score of 2) for a further 10%. Given the anticholinergic burden associated with these medicines, neither would be considered a good choice in older adults, particularly those with pre-existing problems with cognition.

### Other medications with anticholinergic properties

Medications with anticholinergic properties other than those already discussed were prescribed for 16% of the patients in the total patient sample. The three most frequently prescribed medicines in this category were diazepam, lithium and promethazine; these are all psychotropics and as such their use is most likely to have been initiated by mental health services.

### Strengths and limitations of this study

Each older people's mental health service that participated in this audit-based quality improvement programme was invited to submit prescribing data for a random sample of eligible patients in their caseload, so any systematic sampling bias is unlikely. The data collected were related to performance against evidence-based practice standards, gathered using a standardised data collection tool and limited to the information available in the clinical records. The generalisability of the findings rests on the large national sample size, with data submitted by most NHS mental health trusts. However, the findings may not be extrapolated beyond older people's mental health services. The AEC scale was chosen to determine anticholinergic burden as it was developed to identify medications with high central anticholinergic effects and the risk of an adverse effect on cognition.

The findings of this audit of prescribing practice for older adults under the care of mental health services in the UK suggest that such patients are likely to be prescribed multiple medications for psychiatric disorders and comorbid physical disorders. Therefore, prescribers need to be aware of the possibility of high anticholinergic burden, often an inadvertent consequence of the co-prescription of medications of modest anticholinergic activity. They also need to be assiduous in checking for adverse anticholinergic effects, which may range from subtle cognitive impairment to delirium.

The data collected in this study can inform and support interventions by clinicians in older people's services to reduce the anticholinergic burden of patients’ medication regimens and thus potentially minimise the serious adverse consequences of anticholinergic effects. When seeking to reduce a patient's anticholinergic burden, a step-wise approach can be taken: first, consideration of whether the use of a non-pharmacological intervention would be a reasonable alternative to a medication; second, assessing whether intervention early in a prescribing cascade can avoid the use of medication with anticholinergic effects; and, lastly, selection of a medication regimen with a lower anticholinergic burden, potentially guided by the use of a scale such as the AEC, which identifies the level of central anticholinergic activity of relevant medications.

## Data Availability

The aggregated data-set that supports these findings is not openly available. Membership agreements between Prescribing Observatory for Mental Health (POMH) and participating mental health services state that each mental health service owns its own data-set and that this will not be shared by POMH with any third party. POMH is restricted to reporting on analyses based on the aggregated national data-set.
